# Pidotimod alleviated experimental autoimmune encephalomyelitis by regulating the balance of splenic lymphocytes

**DOI:** 10.1186/s12865-025-00736-1

**Published:** 2025-07-21

**Authors:** Yanping Wang, Sifan Zhang, Anqi Li, Ping Zhao, Xiaoru Ma, Xiyu Zhang, Junfeng Wu, Zhixin Qiao, Chao Wang, Xiujuan Lang, Xijun Liu, Bo Sun, Hulun Li, Yumei Liu

**Affiliations:** 1 Department of Neurobiology, School of Basic Medical Sciences, Harbin Medical University, Harbin, Heilongjiang, 150081 PR China; 2 The Key Laboratory of Preservation of Human Genetic Resources and Disease Control in China, Harbin Medical University, Ministry of Education, Harbin, Heilongjiang, 150081 PR China; 3 The Key Laboratory of Myocardial Ischemia, Harbin Medical University, Ministry of Education, Harbin, Heilongjiang, 150081 PR China

**Keywords:** Pidotimod, Multiple sclerosis, Experimental autoimmune encephalomyelitis, Splenic lymphocytes

## Abstract

**Objective:**

To examine whether pidotimod affects the progression and severity of experimental autoimmune encephalomyelitis (EAE), a classic animal model of multiple sclerosis (MS), the balance of splenic lymphocytes in pidotimod-treated and untreated EAE mice was examined.

**Methods:**

C57BL/6J mice were immunized by subcutaneous injection of an emulsion containing MOG35-55, with subsequent monitoring of their general condition and clinical scores following treatment with pidotimod or saline solution (vehicle control). Hematoxylin and eosin (H&E) staining, along with flow cytometry (FCM), was employed to evaluate leukocyte infiltration, while FluoroMyelin™ Green staining was utilized to assess axonal demyelination in the central nervous system (CNS). Additionally, FCM was conducted to investigate the effects of pidotimod on splenic lymphocytes both in vitro and in vivo during the peak stage of EAE.

**Results:**

Compared to the vehicle control, pidotimod treatment significantly reduced the clinical scores, decreased leukocyte infiltration in the spinal cord and brain, and suppressed demyelination in the spinal cord. Furthermore, pidotimod treatment markedly increased the populations of CD4^+^ CD25^+^ Foxp3^+^ regulatory T cells (Tregs) and CD8^+^ Foxp3^+^ Tregs, while decreasing the numbers of CD4^+^ IFN-γ^+^ helper T cells (Th1), CD4^+^ IL-17^+^ helper T cells (Th17), and CD8^+^ IL-17^+^ cytotoxic T cells (Tc17) in the spleen during the peak stage of EAE both in vitro and in vivo. Additionally, pidotimod treatment significantly diminished the population of B220^+^ TNF-α^+^ B cells in the spleen at the peak stage of EAE both in vitro and in vivo.

**Conclusions:**

The present study preliminarily explored the effects and potential immunomodulator mechanisms of pidotimod in treating EAE mice. Results indicated that pidotimod treatment decreased the percentages of CD4^+^ IFN-γ^+^ Th1 cells, CD4^+^ IL-17^+^ Th17 cells, CD8^+^ IL-17^+^ Tc17 cells and B220^+^ TNF-α^+^ B cells, while increasing the percentages of CD4^+^ CD25^+^ Foxp3^+^ Tregs and CD8^+^ Foxp3^+^ Tregs in the spleen at the peak stage of EAE. Additionally, pidotimod reduced leukocyte infiltration into the spinal cord and brain, as well as demyelination in the spinal cord. These findings suggest that the neuroprotective effects of pidotimod in EAE mice may be its ability to regulate the balance of splenic lymphocytes.

**Supplementary Information:**

The online version contains supplementary material available at 10.1186/s12865-025-00736-1.

## Introduction

Multiple sclerosis (MS) is a chronic autoimmune disease characterized by the infiltration of pathogenic inflammatory cells into the central nervous system (CNS), resulting in focal inflammation, demyelination, axonal damage, and neuronal injury [1, 2]. In recent years, the global incidence and prevalence of MS have risen markedly, with over 2.5 million individuals affected worldwide [3]. To date, several treatments for MS have been approved by the Food and Drug Administration of the USA. Despite the availability of various therapeutic approaches, many have yielded unsatisfactory results in reversing the progression of MS due to its complex etiology, highlighting the need for further drug discovery efforts [4]. Experimental autoimmune encephalomyelitis (EAE) in mice serves as a well-established model for MS research and drug discovery. This model effectively mimics key features of MS and is commonly utilized to investigate the disease’s pathogenesis [5, 6].

Antigen-specific T lymphocytes play crucial roles in the progression of MS and EAE [7]. B lymphocytes also have significant physiological functions in MS [8]. These pathogenic inflammatory cells migrate to the CNS, where they are reactivated by local antigen-presenting cells, subsequently triggering CNS inflammation. The activated T cells interact with CNS-resident cells, leading to their activation and increased production of pro-inflammatory cytokines and chemokines. This cascade results in secondary T-cell activation, monocyte recruitment, and widespread CNS inflammation [1]. CD4^+^ T cells have traditionally been considered to play a predominant role throughout MS. During disease progression, various subsets of T lymphocytes exhibit contrasting immune functions. For instance, helper T cells (Th1 or Th17) enhance CNS inflammation by secreting several proinflammatory cytokines, including interferon-gamma (IFN-γ), interleukin-17 (IL-17), and granulocyte macrophage colony stimulating factor (GM-CSF). In contrast, IL-4-producing helper T cells (Th2) cells can mitigate this inflammatory response [9, 10]. Additionally, regulatory T cells (Tregs) suppress the inflammatory cascade by limiting the activation, proliferation, survival, and proinflammatory functions of various immune cells. Multiple lines of evidence suggest that Tregs play an immunosuppressive role in regulating the immune response through various mechanisms in MS [11]. Dysfunction of Tregs and altered cytokine production may facilitate the entry of proinflammatory autoreactive T lymphocytes into the CNS, thereby promoting disease progression [12]. Targeting T lymphocyte subsets to regulate the immune response has the potential to alleviate the progression of EAE in mice. In MS lesions, CD8^+^ T cells outnumber CD4^+^ cells and are associated with disease progression. CNS-infiltrating CD8^+^ T cells, which are specific for neuronal antigens, directly induce axonal and neuronal injury, contributing to cumulative neurological disability in MS patients [13]. Moreover, increasing evidence indicates that B cells play a significant role in the pathogenesis of MS. Notably, the effectiveness of anti-CD20 therapies underscores the importance of B cells in MS patients [14, 15]. Therefore, regulating different immune cell subsets is crucial for developing effective treatments for MS.

Pidotimod is a synthetic dipeptide with biological and immunomodulatory properties that has been widely used for the treatment and prevention of recurrent respiratory tract infections. In adults, pidotimod is used to prevent and treat acute infectious exacerbations of chronic bronchitis and chronic obstructive pulmonary disease [16, 17]. Pidotimod is proved to be able to ameliorate both innate and adaptive immunity and enhances the immune system. Several studies have demonstrated the critical role of pidotimod in regulating T-cell subsets in the context of infections. For instance, in children with infectious mononucleosis, treatment with pidotimod resulted in decreased numbers of CD3^+^ and CD8^+^ cells, alongside increased levels of CD4^+^ cells and elevated CD4^+^/CD8^+^ ratios [18]. In an ovalbumin-induced allergic asthma model, pidotimod treatment resulted in an enhanced Th2 cell immune response [19]. Nevertheless, the effects of pidotimod on immunomodulator in neuroinflammatory disease EAE remain uncertain. Specifically, it is not yet known whether pidotimod can influence EAE mice through its immunomodulatory function and what role it may play in regulating the balance of immune cells.

In this study, we found that pidotimod treatment alleviated the clinical symptoms of EAE mice. Additionally, pidotimod treatment showed immunomodulator functions by reducing the proportions of IFN-γ-producing CD4^+^ T cells, IL-17-producing CD4^+^ and CD8^+^ T cells, as well as TNF-α-producing B cells, while increasing the proportions of CD4^+^ and CD8^+^ Tregs in the spleens of mice at the peak stage of EAE. Our findings demonstrate that pidotimod has neuroprotective effects against EAE, with its mechanism of action involving the regulation of immune cell balance in the spleen.

## Materials and methods

### Mice

Wild-type female C57BL/6J mice (16–18 g) were obtained from Huafukang Biotechnology Company (Beijing, China). All mice were housed under specific-pathogen-free conditions at a temperature of 23 ± 2 °C, with a relative humidity of 52 ± 2%, and maintained on a 12 h light/12 h dark cycle. Standard mouse food and water were available ad libitum. Health status monitoring, including weight loss assessments and clinical score evaluations, was performed for all mice throughout the experimental protocols. The animal studies received approval from the ethics committee for animal experimentation at Harbin Medical University, and care was taken to minimize the suffering of the mice as much as possible.

### Induction, treatment and evaluation of EAE mice

The EAE model mice received subcutaneous injections of 250 µg of myelin oligodendrocyte glycoprotein (MOG_35–55_) (MEVGWYRSPFSRVVHLYRNGK) (Wuhan Bioyears Gene Biotechnology Inc., China) emulsified in complete Freund’s adjuvant (CFA) (Sigma, St. Louis, MO, USA), which contained 4 mg/ml *Mycobacterium tuberculosis* H37Ra (BD Biosciences, San Jose, CA, USA). Subsequently, 250 ng pertussis toxin (List Biological Laboratories, Inc., Campbell, CA, USA) was injected via the tail vein on day 0 and day 2 after MOG_35−55_ immunization. The CFA control group followed the same procedures, except that MOG_35−55_ was not included.

Pidotimod was purchased from Puli Chemical Industries Company (Shandong, China). The EAE-Veh group (administered saline solution) and the EAE-Pidotimod group (receiving pidotimod at 140 mg/kg) each consisted of 9 mice, and they were used for a 21-day behavioral analysis. The CFA group, EAE-Veh group and the EAE-Pidotimod group each consisted of 3 mice, and they were treated for 15 days for the detection of CD4^+^ T cells. The EAE-Veh group and the EAE-Pidotimod group each consisted of 5 mice, and they were treated for 15 days for the detection of CD8^+^ T cells, B cells and histological studies. Treatment with pidotimod or saline solution was conducted via oral gavage starting from the first day of disease induction until the last day of the study. The clinical scores and weight loss of the mice were monitored daily after immunization. The clinical assessment of the mice was performed on a scale of 0–5 as follows: 0, no clinical disease; 1, tail paralysis; 2, unsteady gait; 3, bilateral hind limb paralysis; 4, forelimb paralysis; and 5, death.

### Histological studies

The mice were anesthetized and euthanized with 2% sodium pentobarbital solution. The spinal cord was isolated after intracardiac perfusion with phosphate-buffered saline (PBS) containing 4% paraformaldehyde and subsequently embedded in O.C.T. (Sakura, Japan). 10 μm sections were then cut using a cryostat. FluoroMyelin™ green staining (1:300, Invitrogen, F34651) was performed to assess demyelination. Staining was performed for 20 min at room temperature in the dark, followed by PBS washes prior to mounting. And haematoxylin and eosin (H&E) staining (Servicebio, G1005-1) was performed to assess leukocyte infiltration. Images of demyelination were captured using a fluorescence microscope (Axio Observer; Carl Zeiss, Jena, Germany). Images of H&E staining were captured using optical microscope (Olympus). Image analyses were conducted with ImageJ software (NIH, Bethesda, MD, USA).

### Isolation of single-cell suspensions from the brain

After systemic perfusion, the brain was collected, and the brain tissue was ground and digested with 0.2% collagenase type II at 37 °C for 1 h. The resulting cell suspension was passed through a 40 μm cell strainer to obtain a single-cell suspension. The filtered cell suspension was then centrifuged for 30 min at 400×g at 20 °C using 30% Percoll (Cytiva, Little Chalfont, Buckinghamshire, UK). The cell pellets were subsequently collected and washed thoroughly in preparation for flow cytometry.

### Culture of splenic lymphocytes with pidotimod in vitro

The mouse spleens were taken out under aseptic conditions and ground on a cell strainer. The cell suspension was transferred to a centrifuge tube, and then the cell strainer was washed with 2 mL of sterile cell PBS. The suspension was collected. Centrifuged at 500×g, 4 °C for 10 min, and the supernatant was discarded. 3–4 mL of red blood cell lysis solution was added to resuspend the cells at room temperature for 5 min. 2 times the volume of lysis solution was added to terminate the reaction at 200×g, 4 °C for 10 min. The supernatant was discarded, and the cells were resuspended in 1 mL of sterile PBS and filtered for counting. The 2 × 10^6^ splenic lymphocytes were cultured in complete RPMI 1640 medium containing 10% FBS, 2-mercaptoethanol (50 µM, Sigma), L-glutamine (2 mM, Sigma), penicillin (100 ug/ml, Sigma), and streptomycin (100 µg/ml, Sigma). Pidotimod (10 µg/ml) or PBS (as a vehicle control) was added to evaluate its effect on CD4^+^ and CD8^+^ T cells and B cells in vitro, in the presence of phorbol 12-myristate 13-acetate (PMA) (20 ng/ml, Sigma) and ionomycin (20 ng/ml, Sigma). The cells were collected for flow cytometry analysis after 48-h incubation.

### Flow cytometry (FCM)

Flow cytometry was used to evaluate the percentage of immune cells in the spleen and brain. Single-cell suspensions were stimulated with PMA (50 ng/ml, Sigma), ionomycin (1000 ng/ml, Sigma), and brefeldin A (1x, BioLegend) for 4–6 h at 37 °C. For surface staining, the cells were stained with surface markers for 30 min at 4 °C in the dark. After surface staining, the cells were fixed and permeabilized using fixation/permeabilization solution (BD Biosciences, San Jose, CA, USA) for 30 min at 4 °C in the dark, and then intracellular cytokine staining was conducted. For Foxp3 staining, the Foxp3/Transcription Factor Fixation/Permeabilization Concentrate and Fixation/Permeabilization Solution Diluent (BD Biosciences, San Jose, CA, USA) were applied for 30 min at 4 °C in the dark, and then Foxp3 staining was conducted.

The cells were analysed with a FACSVerse flow cytometer (BD Biosciences), and the data were obtained with FlowJo software. Gating strategies for identifying live single cells, T-cell and B-cell subsets, and cytokine-positive populations were applied using sequential FSC/SSC gating, doublet exclusion (FSC-H vs. FSC-A), and live-cell gating. Full gating hierarchies are illustrated in Supplementary Figs. 1–3. The following antibodies were used:

- PerCP-cyanine5.5-conjugated anti-mouse CD4 antibody (1:100, BioLegend, 100434).
- FITC-conjugated anti-mouse CD4 antibody (1:100, BioLegend, 100510).
- FITC-conjugated anti-mouse CD45 antibody (1:100, BioLegend, 103108).
- PE-conjugated anti-mouse/human CD11b antibody (1:100, BioLegend, 101207).
- FITC-conjugated anti-mouse CD8a antibody (1:100, BioLegend, 100706).
- PE-conjugated anti-mouse CD25 antibody (1:100, eBioscience, 12-0251-83).
- PE-Cy7-conjugated anti-mouse IFN-γ antibody (1:50, eBioscience, 25-7311-82).
- APC-conjugated anti-mouse IFN-γ antibody (1:100, BioLegend, 505809).
- PE-conjugated anti-mouse IL-4 antibody (1:50, BioLegend, 504104).
- APC-conjugated anti-mouse IL-17 A antibody (1:50, BioLegend, 506916).
- APC-conjugated anti-mouse/rat Foxp3 antibody (1:50, eBioscience, 17-5773-82).
- PerCP-Cy5.5-conjugated anti-mouse/human CD45R/B220 antibody (1:100, BioLegend, 103236).
- APC-conjugated anti-mouse IL-6 antibody (1:100, BioLegend, 504508).
- APC-conjugated anti-mouse IL-10 antibody (1:100, BioLegend, 505010).
- APC-conjugated anti-mouse TNF-α antibody (1:100, BioLegend, 506307).
- PE-conjugated anti-mouse GM-CSF antibody (1:100, BioLegend, 505406).

### CCK-8 assay

At the peak of the EAE mice, the spleens were isolated from EAE mice. A total of 2 × 10^6^ splenic lymphocytes were incubated with different concentrations of pidotimod for 48 h. The Cell Counting Kit-8 (CCK8) (abs50003; Shanghai, China) was added into the medium, and the absorbance values were detected at 450 nm after 4 h by an ELISA plate reader (Bio-Rad Laboratories, Hercules, CA, USA).

### Statistical analysis

The normality of the distribution of the data was assessed via the Shapiro-Wilk test (α = 0.05). The assessment of the variance homogeneity of the data was carried out via the F test to compare variances for two groups and the Brown-Forsythe test for multiple groups. When the data were normally distributed, parametric analyses such as unpaired t tests, one-way ANOVA or two-way ANOVA coupled with Tukey post hoc tests, and Brown-Forsythe and Welch ANOVA coupled with Holm-Sidak post hoc tests were used. Where the data were not normally distributed, nonparametric analyses such as the Mann-Whitney U test and the Kruskal-Wallis test coupled with Dunn’s post hoc test were used. The data analyses were performed via GraphPad Prism 8.0. The data are displayed as the means ± SD. *p < 0.05 was considered statistically significant.

## Results

### Pidotimod treatment ameliorated the clinical severity in EAE mice

**Fig. 1:**
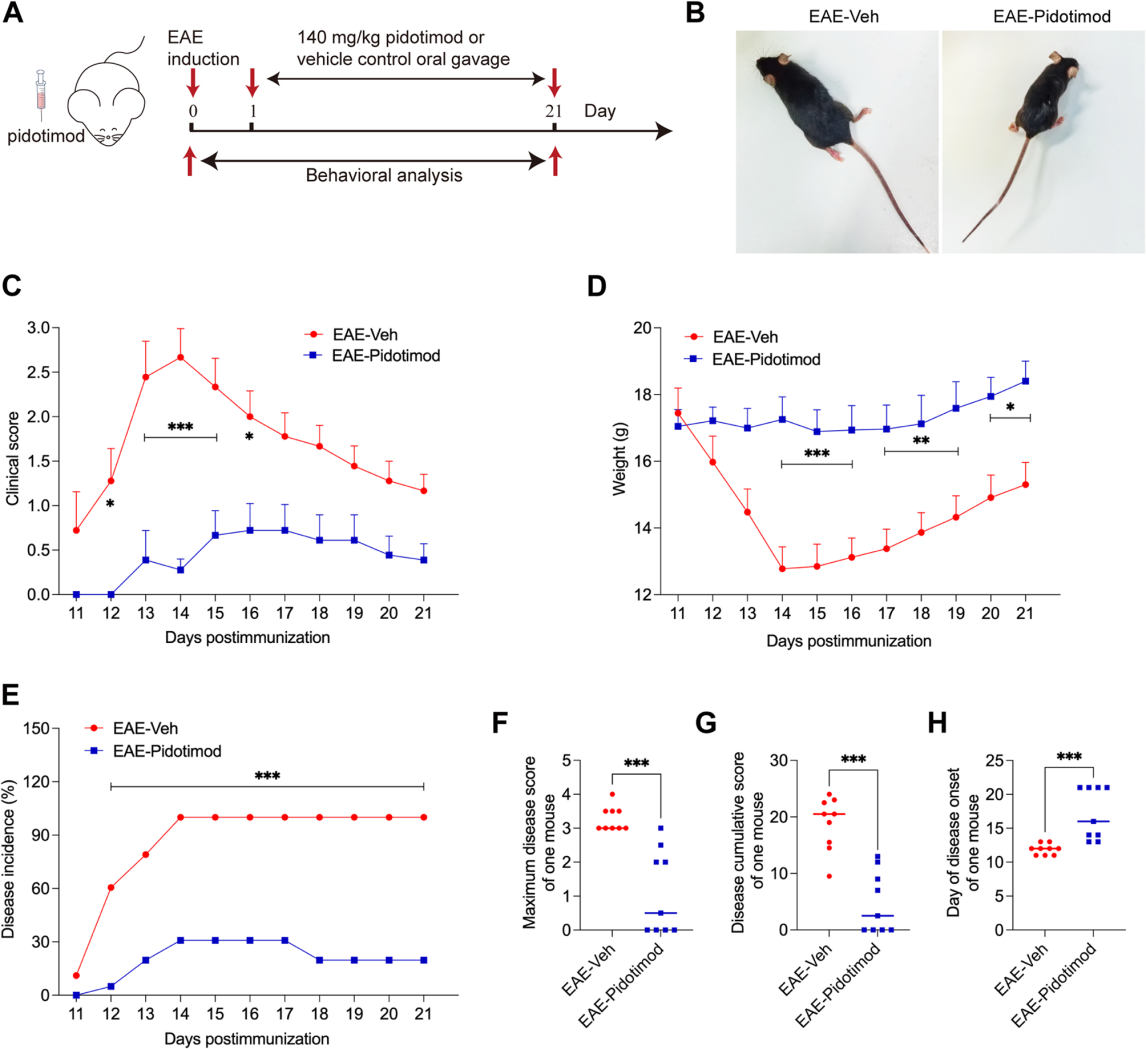
Pidotimod treatment ameliorated the clinical severity in EAE mice (**A**) Schematic diagram of the treatment of EAE model mice with pidotimod or vehicle control. **B**, **C** clinical scores and (**D**) relative weights of mice in the EAE-Veh and EAE-Pidotimod groups. **E** incidence of disease, (**F**) maximal clinical score, (**G**) cumulative clinical score, and (**H**) disease onset were recorded during treatment. The results are expressed as the mean ± SD. *p < 0.05, **p < 0.01, ***p < 0.001. *n* = 9. Statistical analyses were performed via two-way ANOVA (**C**-**E**) or Mann-Whitney U test (**F**-**H**)

To determine the function of the immunomodulator pidotimod in EAE mice, EAE was induced in C57BL/6J mice, which were then treated with either pidotimod or vehicle control. Pidotimod treatment by oral gavage was initiated on the first day of disease induction and continued until the last day of the experimental period. The clinical scores and weights of the mice were evaluated daily following immunization and monitored continuously for 21 days (Fig. 1A). The clinical observations revealed that mice in the EAE-Veh group exhibited bilateral hind limb paralysis. In contrast, the general condition of mice in the EAE-Pidotimod group improved significantly (Fig. 1B). Mice in the EAE-Pidotimod group exhibited lower clinical scores compared to the EAE-Veh group (Fig. 1C). Additionally, pidotimod treatment significantly prevented weight loss in EAE mice (Fig. 1D). Furthermore, the disease incidence (Fig. 1E), maximum clinical score (Fig. 1F), and cumulative clinical score (Fig. 1G) were lower in the EAE-Pidotimod group than in the EAE-Veh group. Disease onset was also delayed in the EAE-Pidotimod group compared to the EAE-Veh group (Fig. 1H). These findings suggest that pidotimod treatment ameliorated the clinical severity of EAE in mice.

### Pidotimod treatment reduced CNS inflammation and demyelination

**Fig. 2:**
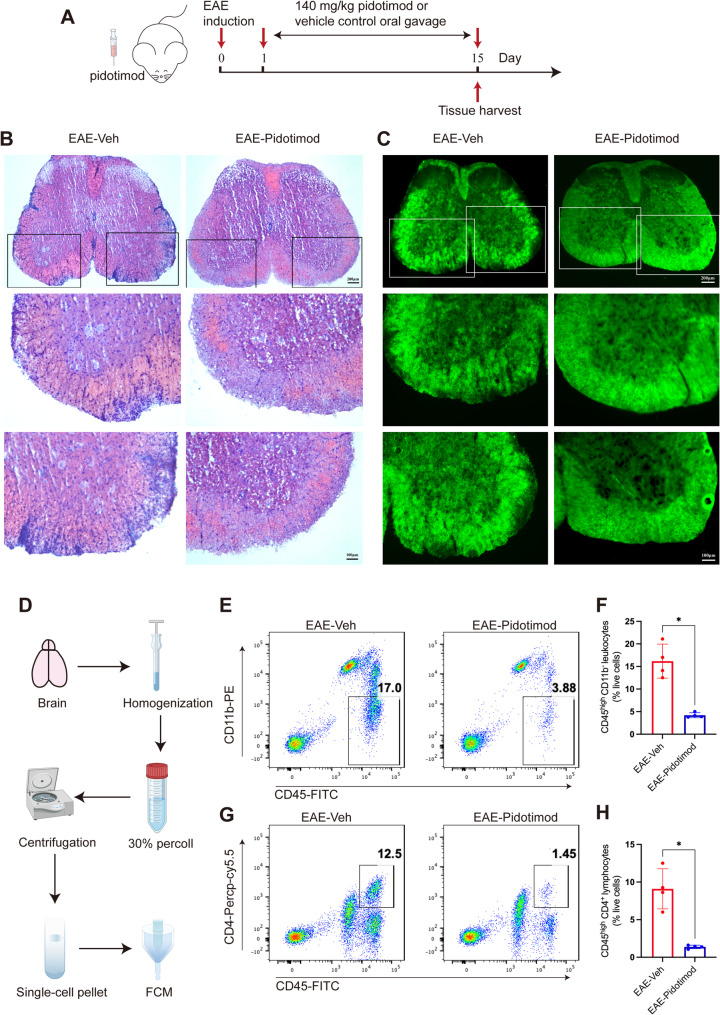
Pidotimod treatment reduced CNS inflammation and demyelination (**A**) Schematic diagram of the treatment of EAE model mice with pidotimod or vehicle control. **B**, **C** Histopathological staining of spinal cord samples from mice in the EAE-Veh and EAE-Pidotimod groups at the peak phase of EAE was performed via H&E (**B**) and FluoroMyelin™ green (**C**) staining, scale bars: 200 μm (low magnification), 100 μm (high magnification). **D** Schematic diagram of single-cell acquisition and FCM. **E** Representative FACS plots of CD45^high^ CD11b^−^ leukocytes isolated from the brains of mice from the EAE-Veh and EAE-Pidotimod groups were shown. **F** Histogram showing the percentage of CD45^high^ CD11b^−^ leukocytes in brain samples from mice in the EAE-Veh and EAE-Pidotimod groups. **G** Representative FACS plots of CD45^high^ CD4^+^ lymphocytes in brain samples from mice in the EAE-Veh and EAE-Pidotimod groups are shown. **H** Histogram showing the percentage of CD45^high^ CD4^+^ lymphocytes in brain samples from mice in the EAE-Veh and EAE-Pidotimod groups. The results are expressed as the mean ± SD. *p < 0.05, *n* = 4. Statistical analyses were performed via Mann-Whitney U test (**F**, **H**)

Leukocyte infiltration into the CNS triggers CNS inflammation. Therefore, at the peak stage of EAE, spinal cords were collected to evaluate leukocyte infiltration and demyelination using H&E and FluoroMyelin™ green staining, respectively (Fig. 2A). H&E staining revealed a significant reduction in the frequency of infiltrating leukocytes in the EAE-Pidotimod treatment group compared to the EAE-Veh group (Fig. 2B). To assess changes in demyelination damage to the spinal cord, the lumbar spinal cord was subjected to FluoroMyelin™ green staining. This reagent stains normal spinal cord tissue green, while demyelinated areas remain uncolored. Consistent with the findings on leukocyte infiltration, pidotimod treatment mitigated demyelination in EAE mice (Fig. 2C). These results suggested that pidotimod alleviated demyelination in EAE mice and reduced leukocyte infiltration into the spinal cord. We subsequently measured leukocyte changes in the CNS after pidotimod treatment via flow cytometry on day 15 postimmunization (Fig. 2D). The percentage of CD45^high^ CD11b^−^ leukocytes infiltrating the brain tissue was significantly lower in the EAE-Pidotimod group compared to the EAE-Veh group (Fig. 2E, F). Additionally, the proportion of CD45^high^ CD4^+^ T lymphocytes in the brain was lower in the EAE-Pidotimod group than in the EAE-Veh group (Fig. 2G, H). Thus, pidotimod treatment hampered the infiltration of CD45^high^ CD11b^−^ leukocytes and CD45^high^ CD4^+^ T lymphocytes into the brain, potentially alleviating EAE progression in the mice.

### Pidotimod regulated the balance of splenic CD4^+^ T-cell subsets in vivo

**Fig. 3:**
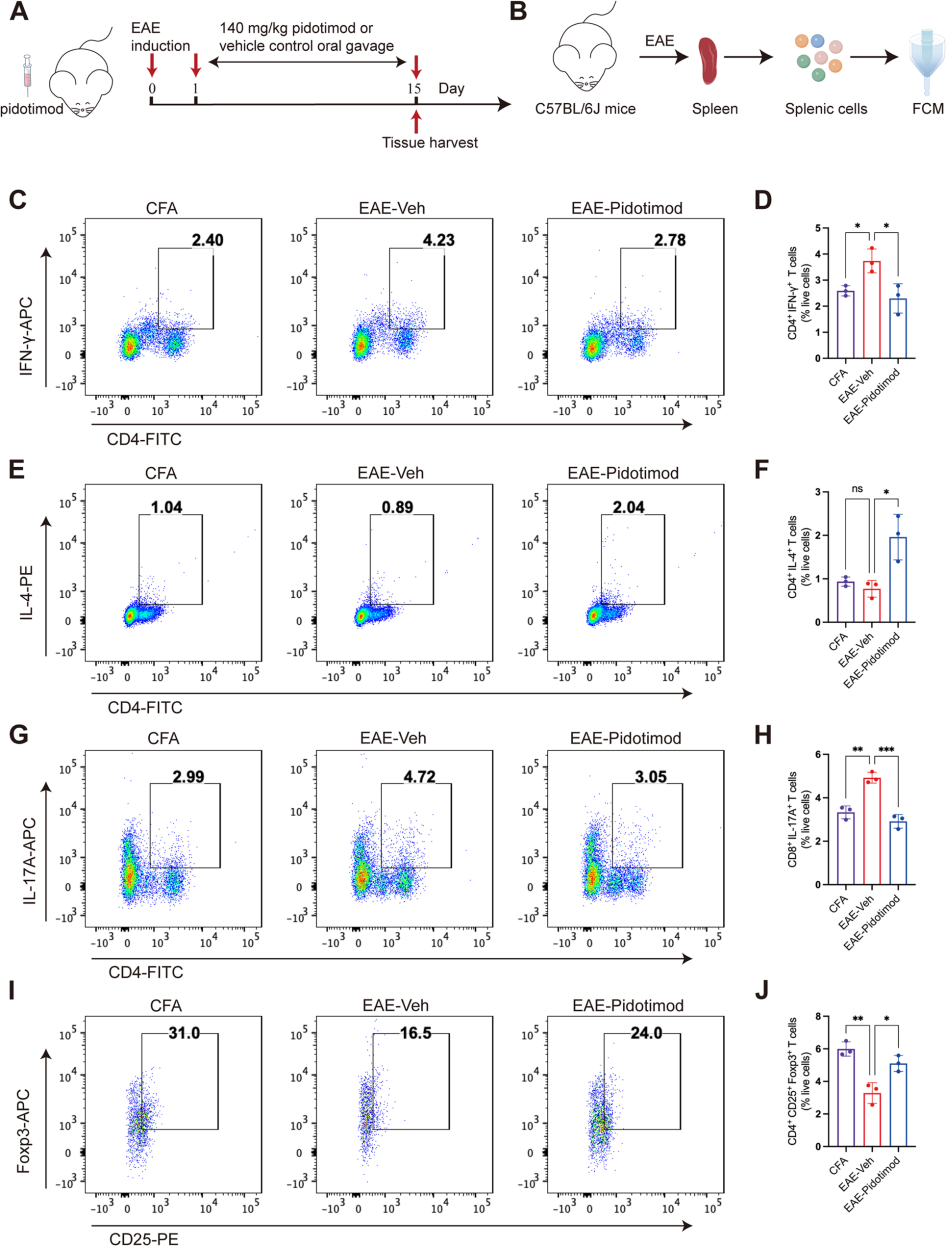
Pidotimod regulated the balance of splenic of CD4^+^ T-cell subsets in vivo (**A**) Schematic diagram of the treatment of EAE model mice with pidotimod or vehicle control. **B** Schematic diagram of single-cell acquisition and FCM. **C**, **E**, **G**, **I** Representative FACS plots depicting the expression of CD4^+^ IFN-γ^+^ Th1 cells (**C**), CD4^+^ IL-4^+^ Th2 cells (**E**), CD4^+^ IL-17^+^ Th17 cells (**G**), and CD4^+^ CD25^+^ Foxp3^+^ Tregs (**I**) in the three groups. **D**, **F**, **H**, **J** Histograms depicting the percentages of CD4^+^ IFN-γ^+^ Th1 cells (**D**), CD4^+^ IL-4^+^ Th2 cells (**F**), CD4^+^ IL-17^+^ Th17 cells (**H**), and CD4^+^ CD25^+^ Foxp3^+^ Tregs (**J**) in the three groups. The results are expressed as the mean ± SD. *p < 0.05, **p < 0.01, ***p < 0.001, ns, nonsignificant (compared with the EAE-Veh group), *n* = 3. Statistical analyses were performed using one-way ANOVA coupled with Tukey’s post hoc test (**D**, **F**, **H**, **J**)

To evaluate the immunomodulatory effects of pidotimod in vivo, we administered the compound by oral gavage and subsequently isolated splenic lymphocytes from three experimental groups: CFA control group, EAE-Veh group, and EAE-pidotimod group for flow cytometry analysis (Fig. 3A, B). Our results revealed significant differences in CD4^+^ T cell subset distribution among the groups (Fig. 3C-J). Notably, pidotimod treatment significantly reduced the proportions of proinflammatory CD4^+^ subsets, including: CD4^+^ IFN-γ^+^ Th1 cells (Fig. 3C, D), CD4^+^ IL-17^+^ Th17 cells (Fig. 3G, H). Conversely, we observed a significant increase in CD4^+^ CD25^+^ Foxp3^+^ Tregs in the EAE-Pidotimod group compared to the EAE-Veh group (Fig. 3I, J). Consistent with our in vitro findings, pidotimod treatment significantly altered the proportion of CD4^+^ IL-4^+^ Th2 cells in EAE mice compared to the EAE-Veh group (Fig. 3E, F). Notably, pidotimod administration restored the frequencies of Th1, Th17, and Treg cells to levels comparable to those in the CFA group (Fig. 3C-J). These in vivo results demonstrate that pidotimod effectively rebalances the Th1/Th17/Treg axis in the spleen at the peak stage of EAE.

### Pidotimod regulated the balance of splenic CD8^+^ T-cell subsets in vivo

**Fig. 4:**
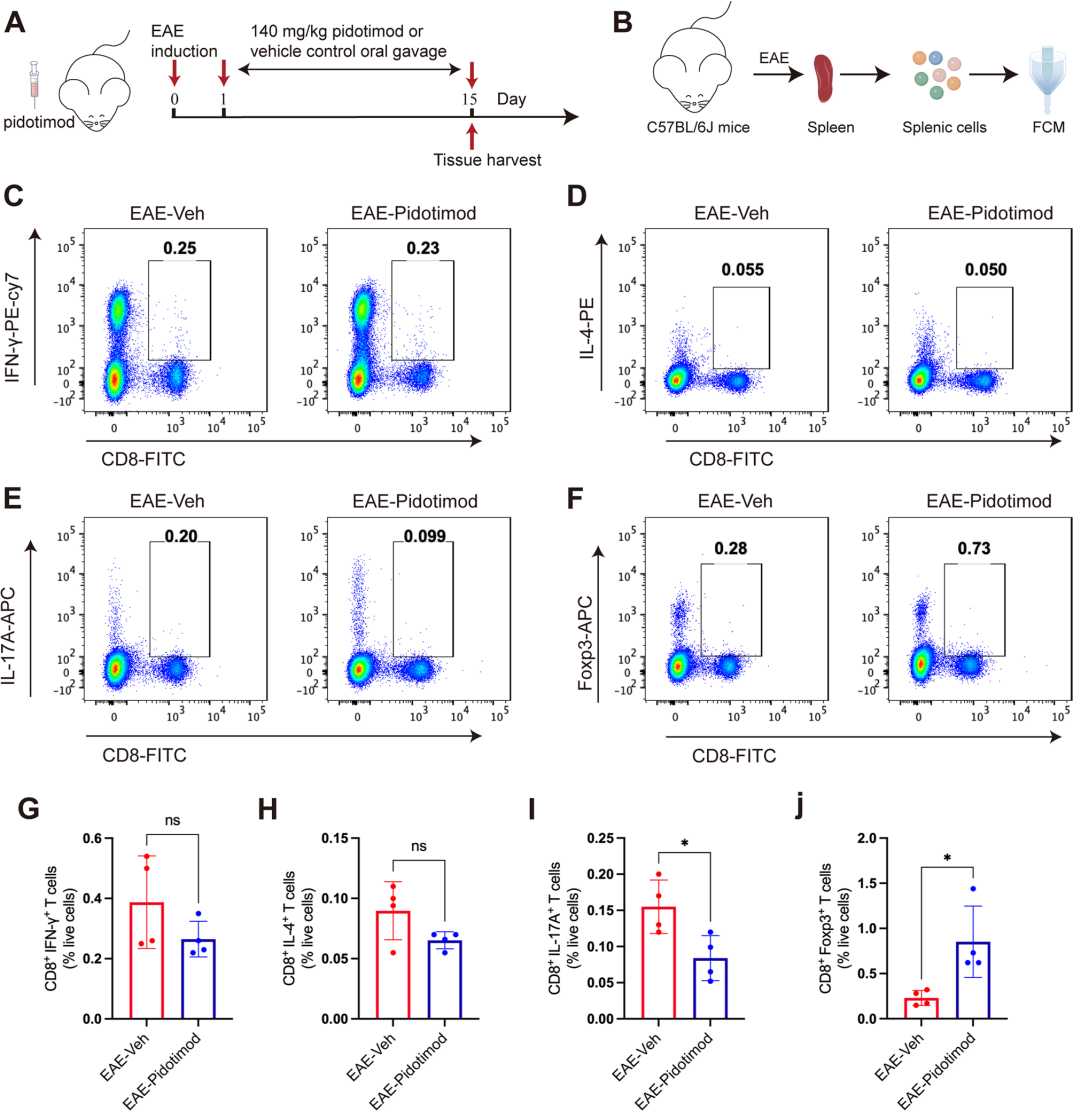
Pidotimod regulated the balance of splenic CD8^+^ T-cell subsets in vivo (**A**) Schematic diagram of the treatment of EAE model mice with pidotimod or vehicle control. **B** Schematic diagram of single-cell acquisition and FCM. **C**-**F** Representative FACS plots depicting the expression of CD8^+^ IFN-γ^+^ Tc1 cells, CD8^+^ IL-4^+^ Tc2 cells, CD8^+^ IL-17^+^ Tc17 cells, and CD8^+^ Foxp3^+^ Tregs. **G**-**J** Histograms depicting the percentages of CD8^+^ IFN-γ^+^ Tc1 cells, CD8^+^ IL-4^+^ Tc2 cells, CD8^+^ IL-17^+^ Tc17 cells, and CD8^+^ Foxp3^+^ Tregs in the EAE-Veh and EAE-Pidotimod groups. The results are expressed as the mean ± SD. *p < 0.05, ns, nonsignificant (compared with the EAE-Veh group), *n* = 4. Statistical analyses were performed via unpaired t-test (**G**, **H**, **I**), Mann-Whitney U test (**J**)

To evaluate the in vivo effects of pidotimod on CD8^+^ T cell populations, we analyzed splenic lymphocytes during peak EAE disease activity (Fig. 4A-B). FCM demonstrated that pidotimod treatment significantly reduced the frequency of proinflammatory CD8^+^ IL-17^+^ Tc17 cells and markedly increased regulatory CD8^+^ Foxp3^+^ Tregs (Fig. 4E, F, I, J). Conversely, we showed no significant effect on CD8^+^ IFN-γ^+^ Tc1 cells (Fig. 4C, G) and CD8^+^ IL-4^+^ Tc2 cells (Fig. 4D, H). These findings indicated that pidotimod promoted the differentiation of CD8^+^ T cells toward an anti-inflammatory Tregs phenotype while suppressing the development of proinflammatory Tc17 cells in the EAE spleen.

### Pidotimod regulated the balance of splenic B-cell subsets in vivo

**Fig. 5:**
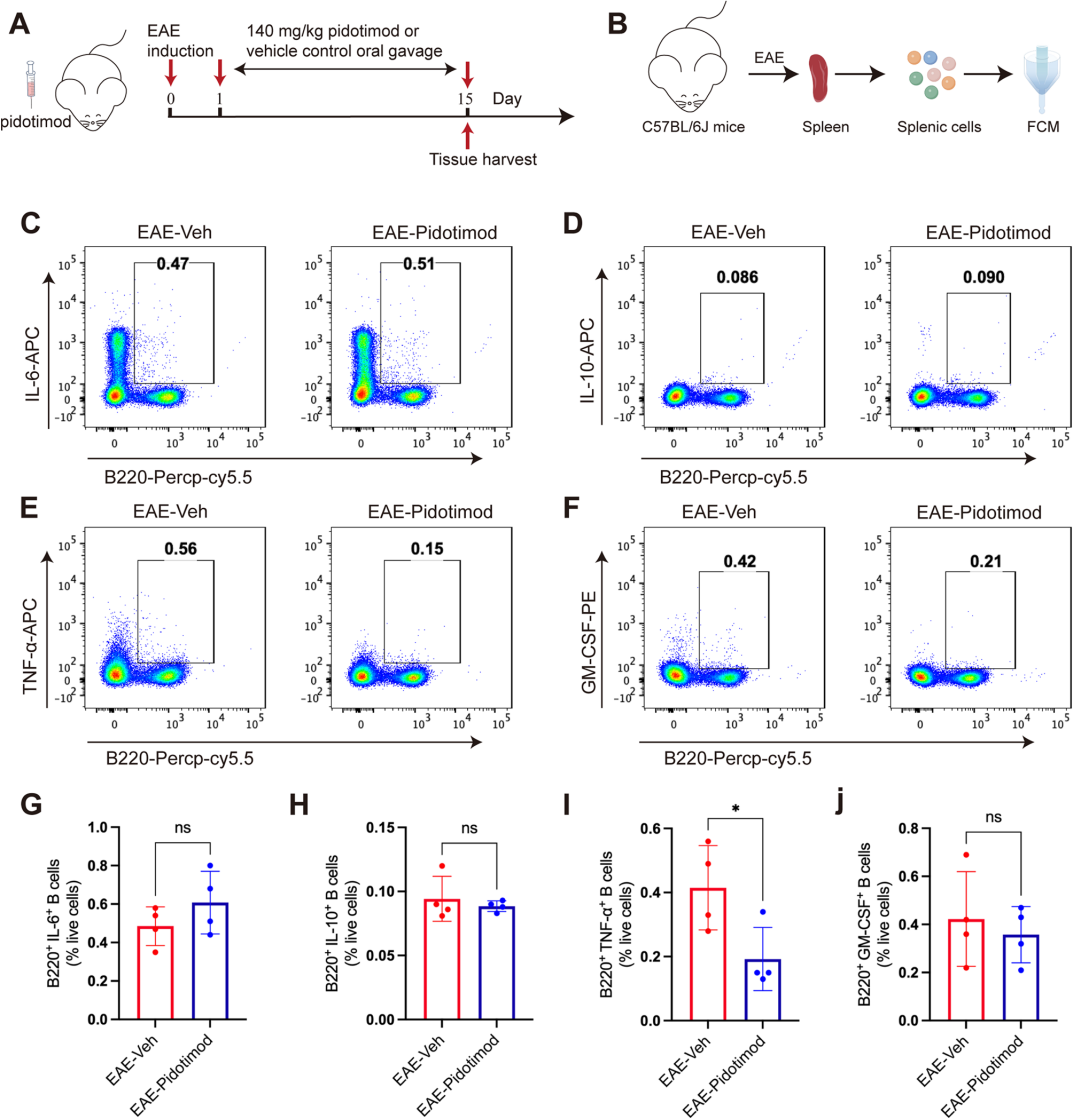
Pidotimod regulated the balance of splenic B-cell subsets in vivo (**A**) Schematic diagram of the treatment of EAE model mice with pidotimod or vehicle control. **B** Schematic diagram of single-cell acquisition and FCM. **C**-**F** Representative FACS plots depicting the numbers of B220^+^ IL-6^+^ B cells, B220^+^ IL-10^+^ B cells, B220^+^ TNF-α^+^ B cells, and B220^+^ GM-SCF^+^ B cells in the spleen at the peak stage in EAE mice. **G**-**J** Histograms depicting the percentages of B220^+^ IL-6^+^ B cells, B220^+^ IL-10^+^ B cells, B220^+^ TNF-α^+^ B cells, and B220^+^ GM-SCF^+^ B cells in the spleen at the peak stage of EAE. The results are expressed as the mean ± SD. *p < 0.05, ns, nonsignificant (compared with the EAE-Veh group); *n* = 4. Statistical analyses were performed via unpaired t test (**G**-**J**)

To evaluate the in vivo effects of pidotimod on B cell populations, we analyzed splenic lymphocytes during peak EAE disease activity (Fig. 5A, B). FCM demonstrated that pidotimod treatment significantly decreased the frequency of proinflammatory TNF-α-producing B cells (Fig. 5E, I). Conversely, we showed no significant alteration in other B cell subsets, including IL-6-producing B cells (B220^+^ IL-6^+^), IL-10-producing B cells (B220^+^ IL-10^+^), GM-CSF-producing B cells (B220^+^ GM-SCF^+^) (Fig. 5C, D, F, G, H, J). These in vivo findings were consistent with our in vitro results and demonstrated that pidotimod specifically reduced the proportion of TNF-α-producing B cells in the EAE spleen.

### Pidotimod regulated the balance of splenic CD4^+^ T-cell subsets in vitro

**Fig. 6:**
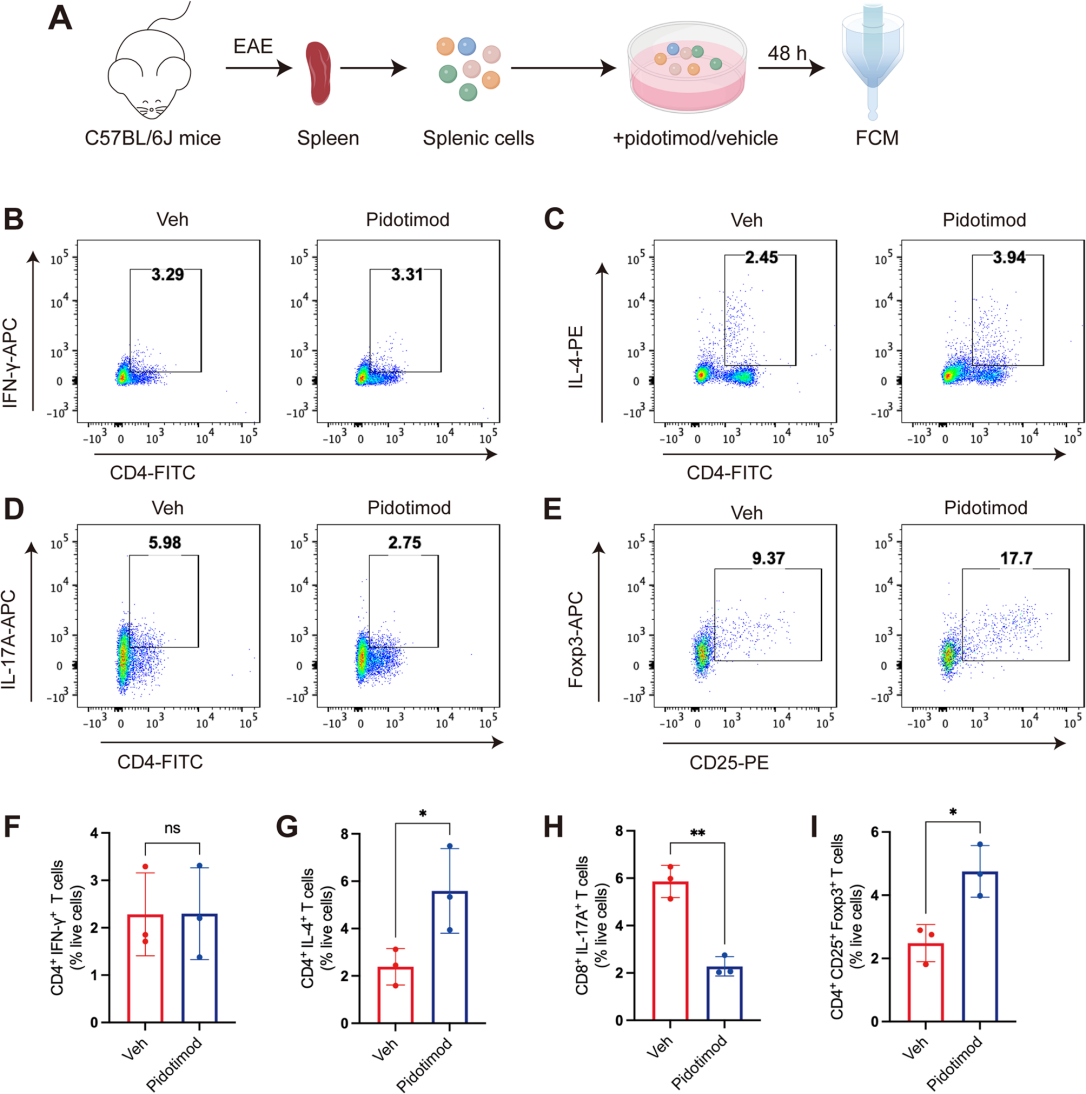
Pidotimod regulated the balance of splenic CD4^+^ T-cell subsets in vitro (**A**) Schematic diagram of single-cell acquisition, coculture, and FCM. **B**-**E** Representative FACS plot depicting the percentages of CD4^+^ IFN-γ^+^ Th1 cells (**B**), CD4^+^ IL-4^+^ Th2 cells (**C**), CD4^+^ IL-17^+^ Th17 cells (**D**), and CD4^+^ CD25^+^ Foxp3^+^ Tregs (**E**) among CD4^+^ T cells in the Veh and Pidotimod groups. **F**-**I** Histogram depicting the percentages of CD4^+^ IFN-γ^+^ Th1 cells, CD4^+^ IL-4^+^ Th2 cells, CD4^+^ IL-17^+^ Th17 cells, and CD4^+^ CD25^+^ Foxp3^+^ Tregs in the Veh and Pidotimod groups. The results are expressed as the mean ± SD. *p < 0.05, **p < 0.01, ns, nonsignificant (compared with the Veh group), *n* = 3. Statistical analyses were performed via unpaired *t* test (**F**-**I**)

Helper T cells are critically involved in the pathogenesis of MS and play a pivotal role in the induction of EAE. To investigate whether pidotimod modulates the polarization of naïve T cells toward proinflammatory or anti-inflammatory subsets, we isolated splenic lymphocytes from EAE mice at the disease peak and cocultured them with pidotimod or PBS (vehicle control) in vitro (Fig. 6A). To determine the cytotoxic of pidotimod on lymphocytes in vitro coculture, the gradient concentrations of pidotimod from 0 µg/ml to 500 µg/ml were evaluated after 48 h coculture. The results of CCK8 assay showed that there were no changes of lymphocytes’ viability in various doses of pidotimod compared to 0 µg/ml control, suggesting that no cytotoxicity of pidotimod was detected on lymphocytes. Therefore, the lowest concentration (10 µg/ml) was used for further studies (Supplementary Fig. 4). Following a 48-h culture of splenic lymphocytes with pidotimod or PBS (vehicle control), the proportions of CD4^+^ T cell subsets were analyzed by FCM. Notably, the pidotimod-treated group exhibited a significantly reduced percentage of proinflammatory CD4^+^ IL-17^+^ Th17 cells compared to the Veh group (Fig. 6D, H). In contrast, the pidotimod-treated group displayed significantly higher proportions of anti-inflammatory CD4^+^ IL-4^+^ Th2 cells and CD4^+^ CD25^+^ Foxp3^+^ Tregs compared to the Veh group (Fig. 6C, E, G, I). However, no statistically significant difference was observed in the percentage of CD4^+^ IFN-γ^+^ Th1 cells between the two groups (Fig. 6B, F). These results suggested that pidotimod regulated the balance between the proinflammatory and anti-inflammatory CD4^+^ T cell subsets in vitro, promoting an anti-inflammatory immune phenotype.

### Pidotimod regulated the balance of splenic CD8^+^ T-cell in vitro

**Fig. 7:**
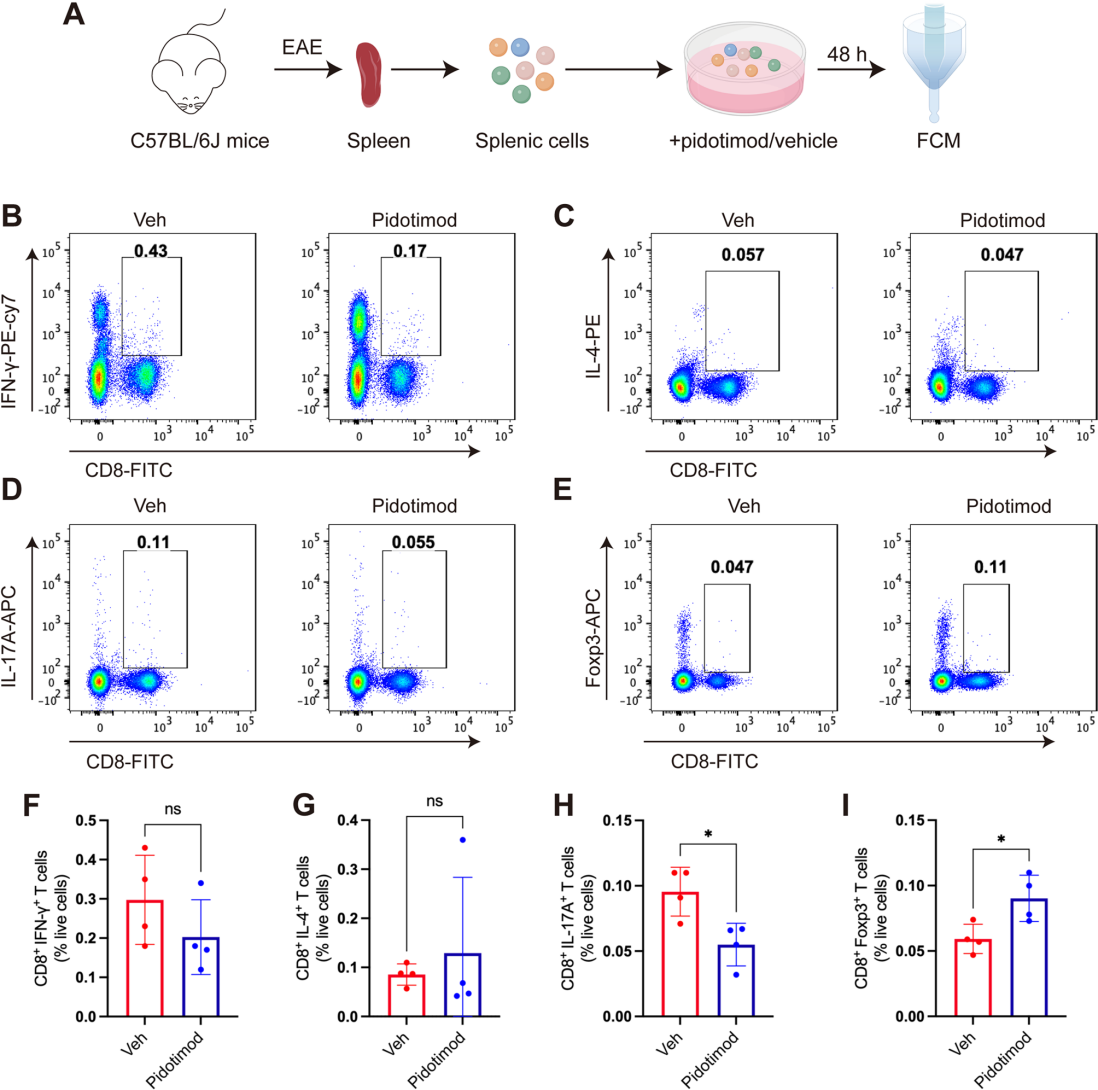
Pidotimod regulated the balance of splenic CD8^+^ T-cell subsets in vitro (**A**) Schematic diagram of single-cell acquisition, coculture, and FCM. **B**-**E** Representative FACS plots depicting the expression of CD8^+^ IFN-γ^+^ Tc1 cells, CD8^+^ IL-4^+^ Tc2 cells, CD8^+^ IL-17^+^ Tc17 cells, and CD8^+^ Foxp3^+^ Tregs. **F**-**I** Histograms depicting the percentages of CD8^+^ IFN-γ^+^ Tc1 cells, CD8^+^ IL-4^+^ Tc2 cells, CD8^+^ IL-17^+^ Tc17 cells, and CD8^+^ Foxp3^+^ Tregs in the Veh and Pidotimod groups. The results are expressed as the mean ± SD. *p < 0.05, ns, nonsignificant (compared with the Veh group), *n* = 4. Statistical analyses were performed via unpaired t test (**F**, **H**, **I**), Mann-Whitney U test (**G**)

While CD4^+^ T cells have been extensively studied in MS pathogenesis, emerging evidence highlights the significant contribution of CD8^+^ cytotoxic T (Tc) cells to disease progression [20]. To investigate whether pidotimod influences CD8^+^ T cell polarization, we conducted parallel in vitro experiments. Splenic lymphocytes isolated from EAE mice at disease peak were cultured with pidotimod or vehicle control for 48 h, followed by FCM of CD8^+^ T cell subsets (Fig. 7A). FCM revealed that pidotimod treatment significantly decreased the frequencies of proinflammatory CD8^+^ subsets CD8^+^ IL-17^+^ Tc17 cells (Fig. 7D, H). Conversely, we observed a significant increase in regulatory CD8^+^ Foxp3^+^ Tregs (Fig. 7E, I). But wo showed no significant effect on CD8^+^ IFN-γ^+^ Tc1 cells and CD8^+^ IL-4^+^ Tc2 cells (Fig. 7B, C, F, G). These findings demonstrate that pidotimod drives CD8^+^ T cell differentiation toward an anti-inflammatory CD8^+^ Tregs phenotype while suppressing proinflammatory Tc1 and Tc17 cell development.

###  Pidotimod regulated the balance of splenic B-cell subsets in vitro

**Fig. 8:**
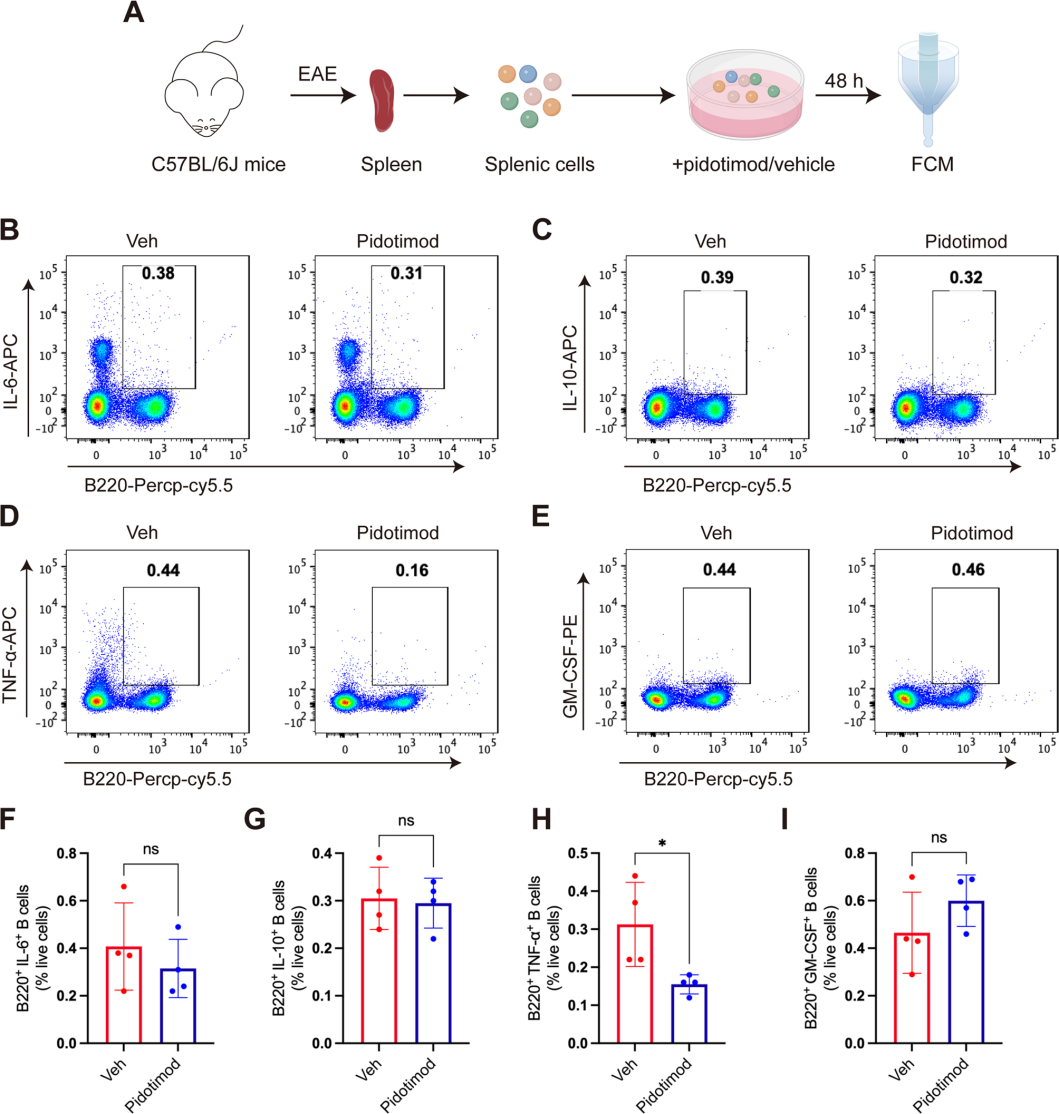
Pidotimod regulated the balance of splenic B-cell subsets in vitro (**A**) Schematic diagram of single-cell acquisition, coculture, and FCM. **B**-**E** Representative FACS plots depicting the expression of B220^+^ IL-6^+^ B cells, B220^+^ IL-10^+^ B cells, B220^+^ TNF-α^+^ B cells, and B220^+^ GM-SCF^+^ B cells after culture with pidotimod or vehicle. **F**-**I** Histograms depicting the percentages of B220^+^ IL-6^+^ B cells, B220^+^ IL-10^+^ B cells, B220^+^ TNF-α^+^ B cells, and B220^+^ GM-SCF^+^ B cells after culture with pidotimod or vehicle. The results are expressed as the mean ± SD. *p < 0.05, ns, nonsignificant (compared with the Veh group), *n* = 4. Statistical analyses were performed via unpaired t-test (**F**-**I**)

Given the emerging role of B cells in MS pathogenesis, we investigated whether pidotimod influences B cell polarization. Splenic lymphocytes were isolated from EAE mice at disease peak and cultured with pidotimod or vehicle control for 48 h (Fig. 8A). FCM demonstrated significant reduction in TNF-α-producing B cells (Fig. 8D, H). Conversely, we showed no significant effects on IL-6-producing B cells (B220^+^ IL-6^+^), IL-10-producing B cells (B220^+^ IL-10^+^), GM-CSF-producing B cells (B220^+^ GM-SCF^+^) (Fig. 8B, C, E, F, G, I). These findings suggested that pidotimod selectively suppressed TNF-α production by B cells without globally altering other cytokine-producing B cell subsets.

## Discussion

MS is an autoimmune disease of the CNS. The characteristic T and B lymphocyte infiltration observed in MS lesions strongly supports an autoimmune basis for disease pathogenesis [15, 21]. The maintenance of immune homeostasis critically depends on the precise balance of lymphocyte subsets. In MS, current evidence supports a model of peripheral activation of T and B lymphocytes followed by CNS invasion. T lymphocytes can be divided into CD4^+^ and CD8^+^ T cells. CD4^+^ T cells, including functionally distinct subsets (Th1, Th17, Treg), have historically been considered the primary mediators throughout the disease course of MS.

Current evidence indicates that the pathogenesis of MS and EAE involves activation and differentiation of myelin-specific CD4^+^ T cells in peripheral lymphoid organs, particularly CD4^+^ IFN-γ^+^ Th1 cells and CD4^+^ IL-17^+^ Th17 cells [22–24]. Tregs are vital for the maintenance of immune homeostasis [12]. Preclinical studies, including data from this investigation, support the therapeutic potential of Treg modulation in many immune-mediated diseases. Furthermore, the Treg/Th17 balance appears to influence disease progression, with a skewed ratio favoring Th17 cells implicated in the transition from acute neuroinflammation to chronic pathology. Dysregulation of the peripheral Treg/Th17 balance aggravates neuroinflammation and neurological deficits in active EAE/MS [25]. Emerging evidence challenges the CD4^+^ exclusivity hypothesis. Recently, CD8^+^ T cells have been increasingly recognized as important contributors, exhibiting cytotoxic potential against neural targets [26]. While CD4^+^ T cells play a key role in initiating MS CD8^+^ T cells emerge as the predominant mediators of CNS damage during relapses and likely contribute to chronic phase pathology. Histopathological analyses of MS patient brain tissue reveal CD8^+^ T cells in close apposition to demyelinated axons. CD8^+^ T cells mediate effector functions through the cytokine production (e.g., IFN-γ, IL-4, TNF-α, and IL-17), or direct cytotoxicity [20]. Emerging evidence further underscores the complexity of CD4^+^/CD8^+^ T-cell interactions in MS. For instance, regulatory CD8^+^ T cells can suppress myelin-specific CD4^+^ T cell activity [27], while proinflammatory cytokines derived from both subsets contribute to MS pathogenesis. Notably, CD8^+^ IL-17^+^ Tc17 cells contribute to the initiation of CNS autoimmunity in murine models and humans by enhancing Th17 cell pathogenicity [28]. The contributions of B cells to disease pathogenesis are increasingly recognized [29]. B cells exhibit dual immunomodulatory functions in T-cell-mediated autoimmune diseases, demonstrating both protective effects through regulatory cytokine secretion (e.g., IL-10) and pathogenic contributions via proinflammatory mediators (including TNF-α, IL-6, and GM-CSF) [30, 31]. Notably, in MS, IFN-γ-producing Th1 cells promote the activation of pathogenic T-bet^+^ B cells [15]. Therapeutic interventions targeting this axis further underscore the critical role of B-T cell crosstalk in driving MS pathogenesis.

Mechanistic studies reveal that pidotimod enhances humoral and cellular immunity, as evidenced by increased serum immunoglobulin levels, CD3^+^ T cells, and CD4^+^ T-cell subtypes. Importantly, clinical trials have established its favorable safety profile, with no significant increase in adverse events [32, 33]. At the molecular level, pidotimod significantly facilitates IL-4-induced M2 macrophage polarization [34] and reduces the number of TNF-α-producing immune cells in adults with community-acquired pneumonia, suggesting its potential as a modulator of inflammatory responses [17]. Pidotimod’s ability to enhance anti-inflammatory cytokines and suppress pro-inflammatory mediators has been documented in systemic diseases [35, 36]. However, contrasting evidence demonstrates that pidotimod can activate dendritic cells (DCs) to secrete substantial quantities of proinflammatory mediators, including MCP-1 and TNF-α, and to drive T-cell proliferation and differentiation towards the Th1 phenotype [37]. Consistent with its proinflammatory potential, pidotimod administration was found to aggravate allergic pulmonary inflammation in an ovalbumin-induced murine asthma model [19]. These divergent findings suggest that pidotimod’s immunomodulatory effects may be context-dependent, varying according to disease pathophysiology. Notably, its impact on neuroinflammatory disorders remains to be elucidated.

**Fig. 9:**
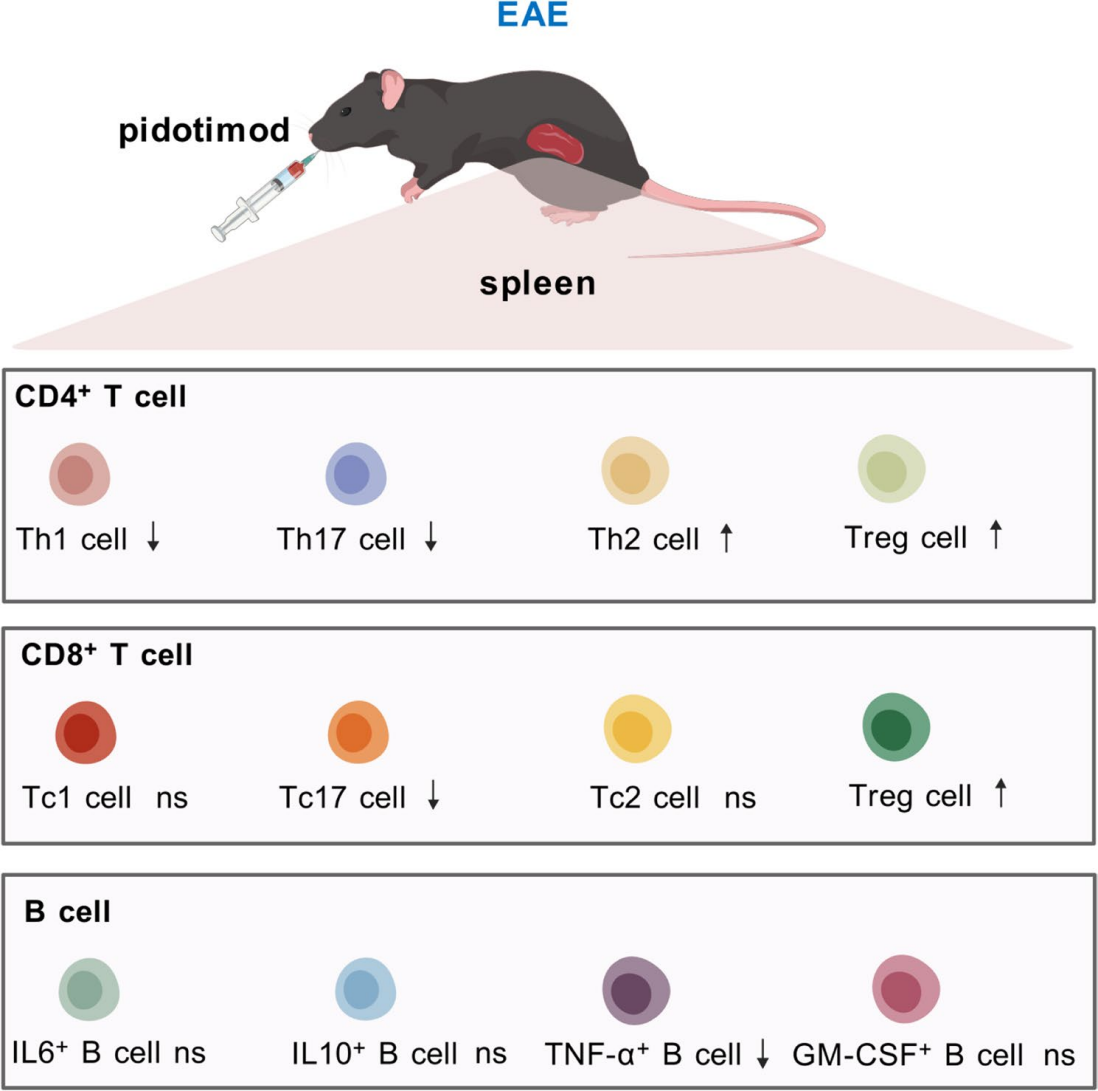
The diagram of the immunomodulatory effects of pidotimod in EAE mice

Given that EAE and MS are characterized by Th1/Th17-driven inflammation, defective Treg function, and macrophage/microglial activation, these immunological actions of pidotimod provide a theoretical and mechanistic basis for its potential benefit in neuroinflammatory models. In this study, we found that pidotimod-treated EAE mice exhibited significant improvements in general condition, as evidenced by reduced cumulative clinical scores. Histopathological analysis further confirmed attenuated inflammatory cell infiltration and diminished demyelination in the spinal cords of pidotimod-treated EAE mice. These findings align with previous reports of EAE amelioration in experimental models, suggesting that pidotimod may hold therapeutic potential for MS [38]. To further investigate the immunomodulatory effects of pidotimod, we analyzed CNS-infiltrating lymphocytes by flow cytometry. The results revealed a significant reduction in the proportions of CD45^high^ CD11b^-^ leukocytes and CD45^high^ CD4^+^ T lymphocytes. Subsequent in vitro and in vivo analyses of CD4^+^ T-cell subsets demonstrated that pidotimod treatment decreased the frequencies of CD4^+^ IFN-γ^+^ Th1 cells and CD4^+^ IL-17^+^ Th17 cells while increasing the proportion of CD4^+^ CD25^+^ Foxp3^+^ Tregs in the spleens of EAE mice. Moreover, pidotimod treatment significantly reduced the proportions of CD8^+^ IL-17^+^ Tc17 cells while elevating CD8^+^ Foxp3^+^ Tregs in the spleen of EAE mice. Additionally, pidotimod treatment markedly decreased the frequency of B220^+^ TNF-α^+^ B cells without affecting other B-cell populations. These results suggest that pidotimod exerts a therapeutic effect on EAE progression by modulating splenic immune responses, thereby restoring the balance between proinflammatory and anti-inflammatory lymphocyte subsets (Fig. 9). Consequently, pidotimod may serve as an immunomodulator reagent in the context of EAE or MS.

Despite the promising findings, our study has some limitations. First, although representative histological images (H&E and FluoroMyelin™ staining) are provided, quantitative analysis of the surface area of inflammatory or demyelinated regions was not performed due to the limited number and resolution of available tissue sections. As such, the histological assessment remains qualitative, based on visual comparison. In future studies, incorporating ImageJ-based quantitative analysis will improve the objectivity and reproducibility of histological evaluation. Additionally, while the study provides the first experimental evidence of pidotimod’s immunomodulatory effects in EAE, further research is needed to determine the mechanistic pathways involved, including its influence on antigen-presenting cells, cytokine signaling cascades, and blood-brain barrier integrity. These findings lay the groundwork for exploring pidotimod as a context-dependent immunomodulatory therapy in neuroinflammatory conditions such as multiple sclerosis.

## Conclusion

This study provides a preliminary investigation into the immunomodulatory effects and potential mechanisms of pidotimod in EAE in mice. Our findings indicated that pidotimod treatment reduced the frequencies of proinflammatory lymphocytes, including IFN-γ-producing CD4^+^ T cells, IL-17-producing CD4^+^ and CD8^+^ T cells, as well as TNF-α-producing B cells, while increasing the proportions of immunoregulatory CD4^+^ and CD8^+^ Tregs in the spleen during the peak phase of EAE.

## Supplementary Information

Supplementary Material 1.

Supplementary Material 2.

## Data Availability

The data supporting the results reported in the article are available on request from the corresponding author upon reasonable request.

## Abbreviations

MS: Multiple sclerosis
EAE: Experimental autoimmune encephalomyelitis
MOG35-55: Myelin oligodendrocyte glycoprotein peptide 35-55
CFA: Complete Freund's adjuvant
CNS: Central nervous system
HE: Hematoxylin and eosin
FCM: Flow cytometry
RT-qPCR: Quantitative real-time polymerase chain reaction
Th1: CD4^+^ IFN-γ^+^ helper T cells
Th2: CD4^+^ IL4^+^ helper T cells
Th17: CD4^+^ IL-17^+^ helper T cells
Tc1: CD8^+^ IFN-γ^+^ cytotoxic T cells
Tc2: CD8^+^ IL-4^+^ cytotoxic T cells
Tc17: CD8^+^ IL-17^+^ cytotoxic T cells
Tregs: Regulatory T cells
