# Supplementary material for: Pidotimod alleviated experimental autoimmune encephalomyelitis by regulating the balance of splenic lymphocytes
Source: BMC Immunol. 2025 Jul 21;26:53. doi: 10.1186/s12865-025-00736-1 (PMC12281762; doi:10.1186/s12865-025-00736-1)
Supplement: Supplementary file 2 — Supplementary Material 2. [file 12865_2025_736_MOESM2_ESM.docx]

**Supplementary data 1**

**
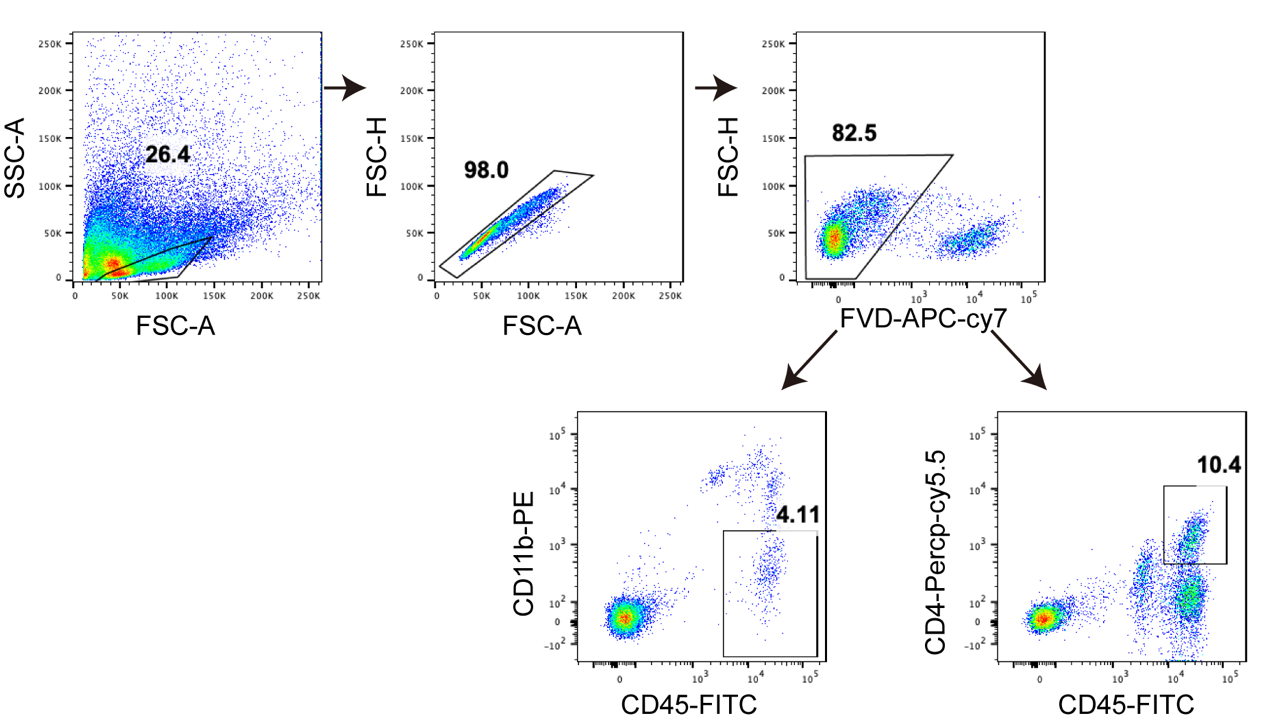
**

Supplementary Fig. 1. The gating strategy of CD45^high^ CD11b^-^ leukocytes and CD45^high^ CD4^+^ lymphocytes.


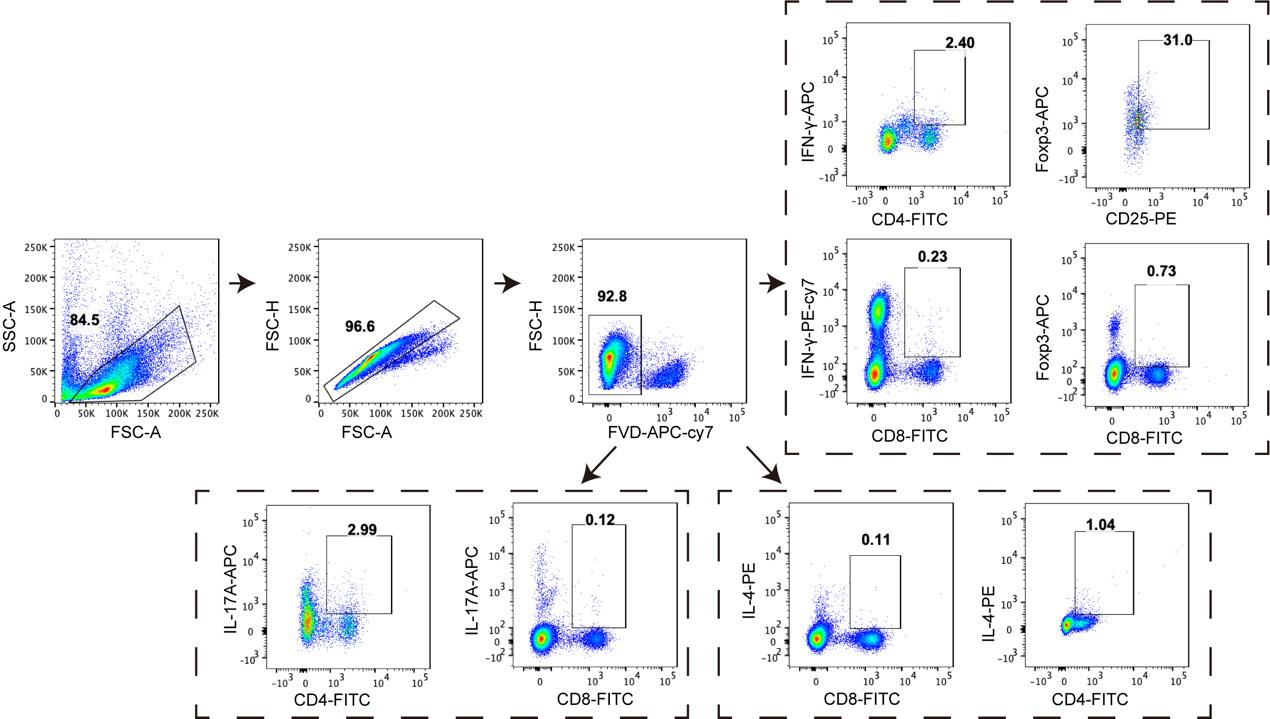


Supplementary Fig. 2. The gating strategy of T lymphocytes.


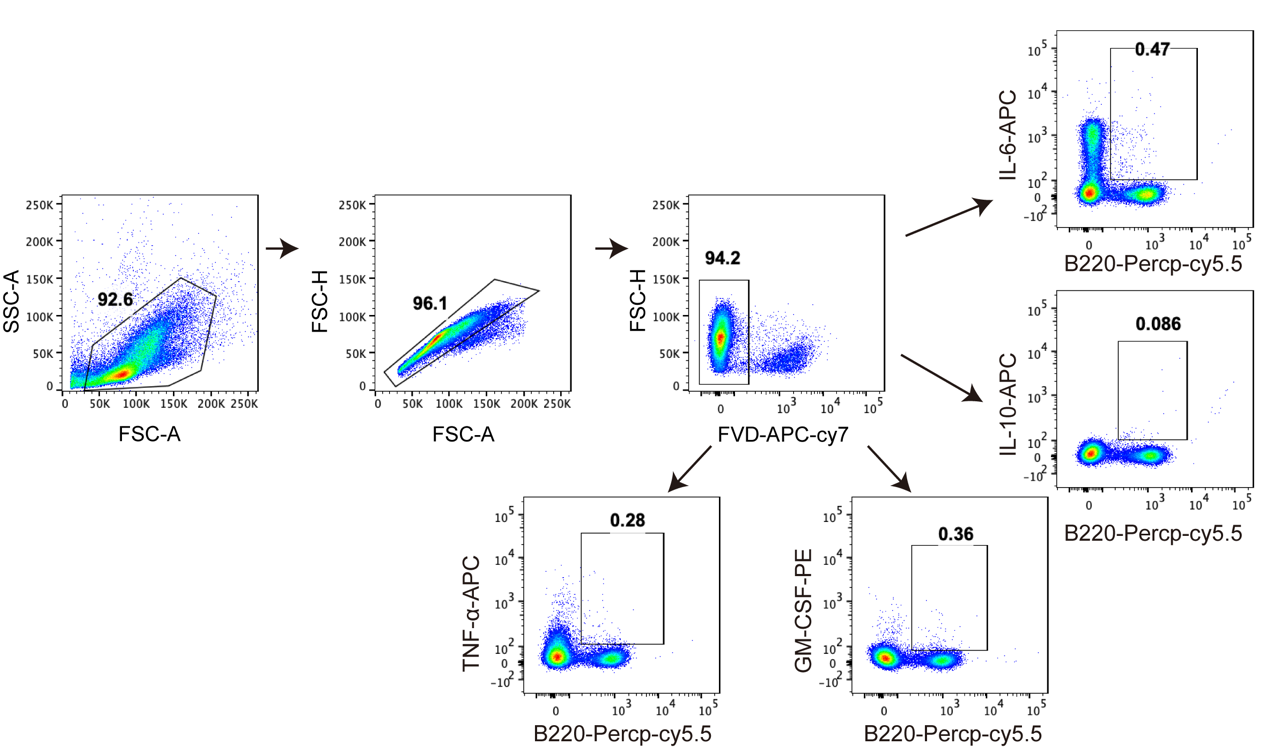


Supplementary Fig. 3. The gating strategy of B lymphocytes.


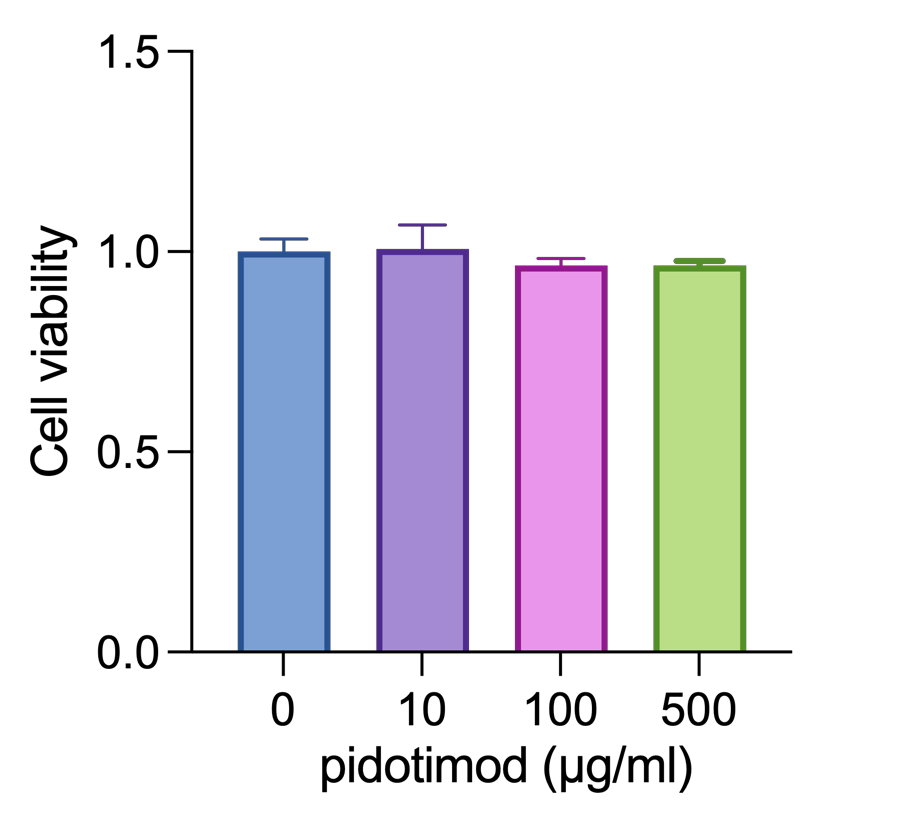


Supplementary Fig. 4. The cell viability was determined by CCK8 assay. The splenic lymphocytes were cultured with different concentrations (10 µg/ml, 100 µg/ml, 500 µg/ml) of pidotimod for 48 h. The CCK8 reagent was added into the medium, and the absorbance values were detected at 450 nm.
